# The carboxylesterase AtCXE12 converts volatile (*Z*)-3-hexenyl acetate to (*Z*)-3-hexenol in Arabidopsis leaves

**DOI:** 10.1093/plphys/kiaf119

**Published:** 2025-03-27

**Authors:** Tristan M Cofer, James H Tumlinson

**Affiliations:** Department of Entomology, Pennsylvania State University, University Park, PA 16803, USA; Institute of Plant Sciences, University of Bern, 3013 Bern, Switzerland; Department of Entomology, Pennsylvania State University, University Park, PA 16803, USA

## Abstract

The green leaf volatiles (GLVs) (*Z*)-3-hexenal, (*Z*)-3-hexenol, and (*Z*)-3-hexenyl acetate play important roles in plant defense, deterring insect herbivores and attracting their natural enemies, while also serving as airborne signaling molecules capable of enhancing defenses in undamaged plant tissues. Almost all plants produce GLVs after wounding, beginning with the formation of (*Z*)-3-hexenal, which is subsequently converted to (*Z*)-3-hexenol and (*Z*)-3-hexenyl acetate. (*Z*)-3-hexenyl acetate can then be taken up by nearby plant tissues where it is predicted to be hydrolyzed to (*Z*)-3-hexenol, a process that is likely to be important in regulating the specific activities of these compounds. However, the enzyme(s) involved in this process and its role in plant defense are largely unknown. Here, we show that Arabidopsis (*Arabidopsis thaliana*) plants rapidly take up (*Z*)-3-hexenyl acetate and convert it to (*Z*)-3-hexenol. Inhibitor and fractionation experiments identified the carboxylesterases Carboxylesterase 5 (AtCXE5) and Carboxylesterase 12 (AtCXE12) as likely contributors to the (*Z*)-3-hexenyl acetate esterase activity in Arabidopsis leaves. Heterologous expression of AtCXE5 and AtCXE12 in *Escherichia coli* revealed that both recombinant enzymes hydrolyze (*Z*)-3-hexenyl acetate to (*Z*)-3-hexenol. Furthermore, assays using T-DNA insertion mutants showed that AtCXE12 significantly contributes to (*Z*)-3-hexenyl acetate hydrolysis in Arabidopsis. Lastly, we found that leaves from several other plant species possess (*Z*)-3-hexenyl acetate esterase activity, suggesting a conserved mechanism for GLV metabolism among plants. Overall, our study provides a better understanding of the biosynthesis and conversion dynamics of GLVs, which is necessary for unraveling the potential functions of these compounds.

## Teaser

A carboxylesterase hydrolyzes the green leaf volatile (Z)-3-hexenyl acetate in Arabidopsis leaves, and similar activity in leaf extracts from several other plant species suggests a conserved mechanism for green leaf volatile metabolism.

## Introduction

In response to wounding and insect attack, nearly all plants produce the green leaf volatiles (GLVs) (*Z*)-3-hexenal, (*Z*)-3-hexenol, and (*Z*)-3-hexenyl acetate (Ameye et al. 2018; Engelberth and Engelberth 2020; Matsui and Engelberth 2022). These compounds can act as a direct defense by exerting toxic or repellent effects on insect herbivores (Hildebrand et al. 1993; Vancanneyt et al. 2001; Allmann et al. 2013) and as an indirect defense by attracting the natural enemies of an attacking herbivore (Shiojiri et al. 2006; Halitschke et al. 2008; Allmann and Baldwin 2010). In addition, GLVs can serve as airborne signaling molecules capable of enhancing defenses in undamaged plant tissues prior to herbivore attack (Bate and Rothstein 1998; Engelberth et al. 2004; Frost et al. 2008; Ameye et al. 2015; Hu et al. 2019; Wang et al. 2023; Waterman et al. 2024).

The production of GLVs begins almost immediately after wounding, starting with the formation of (*Z*)-3-hexenal at the wound site (Fall et al. 1999; D’Auria et al. 2007; Brilli et al. 2011). (*Z*)-3-hexenal can subsequently diffuse into unwounded plant tissues, where it is rapidly converted to (*Z*)-3-hexenol (Matsui et al. 2012). (*Z*)-3-hexenol can then be glycosylated to produce various (*Z*)-3-hexenyl glycosides (Sugimoto et al. 2015, 2021), including (*Z*)-3-hexenyl vicianoside, a compound known to inhibit the growth of some insect herbivores (Sugimoto et al. 2014). Alternatively, (*Z*)-3-hexenol can be converted to (*Z*)-3-hexenyl acetate, which is more volatile than (*Z*)-3-hexenol and therefore more likely to partition from the aqueous cellular environment into the surrounding atmosphere (Henry's law constants for (*Z*)-3-hexenyl acetate and (*Z*)-3-hexenol are ∼1 m atm-¹ and ∼25 m atm-¹, respectively; Fall et al. 1999). Similar to (*Z*)-3-hexenal and (*Z*)-3-hexenol, exogenous (*Z*)-3-hexenyl acetate can be taken up by plants and directly induce or otherwise prime anti-herbivore defenses (Engelberth et al. 2004; Frost et al. 2008; Hu et al. 2019; Wang et al. 2023). While the metabolic fate of (*Z*)-3-hexenyl acetate taken up by plants is unclear, some plants exposed to synthetic (*Z*)-3-hexenyl acetate have been reported to accumulate (*Z*)-3-hexenyl glycosides (Ameye et al. 2020; Sugimoto et al. 2021), suggesting that (*Z*)-3-hexenyl acetate may be hydrolyzed to (*Z*)-3-hexenol and then glycosylated in plant tissues.

The biosynthesis of GLVs is initiated by 13-lipoxygenase, a nonheme iron-containing dioxygenase that adds molecular oxygen to the C13 position of α-linolenic acid (Liavonchanka and Feussner 2006). The resulting 13-hydroperoxy fatty acid is next cleaved by hydroperoxide lyase to produce (*Z*)-3-hexenal (Matsui et al. 1996), which is then reduced to (*Z*)-3-hexenol by a NADP(H)-dependent oxidoreductase (Tanaka et al. 2018). (*Z*)-3-hexenol can thereafter be glycosylated by uridine diphosphate-dependent glycosyltransferases (Jing et al. 2019; Sugimoto et al. 2023) or further converted to (*Z*)-3-hexenyl acetate by a BAHD acetyltransferase (D'Auria et al. 2007). Carboxylesterases (CXEs) capable of hydrolyzing volatile esters such as (*Z*)-3-hexenyl acetate have been identified in the fruits of several plant species (Souleyre et al. 2011; Goulet et al. 2012; Cao et al. 2019; Martínez-Rivas et al. 2022). However, it remains unknown whether similar esterases are present and active in plant leaves.

In this study, we aimed (i) to determine whether exogenous (*Z*)-3-hexenyl acetate is taken up and converted to (*Z*)-3-hexenol by Arabidopsis (*Arabidopsis thaliana*) plants and, if so, (ii) to identify the enzyme(s) responsible for this conversion. To accomplish this, we used the Arabidopsis ecotype Columbia (Col-0) as our focal plant, which does not produce endogenous GLVs due to a natural mutation in *hydroperoxide lyase* (Duan et al. 2005). We found that Arabidopsis plants accumulate (*Z*)-3-hexenol after exposure to synthetic (*Z*)-3-hexenyl acetate and that crude leaf extracts from Arabidopsis possess (*Z*)-3-hexenyl acetate esterase activity. Inhibitor and fractionation experiments identified the carboxylesterases Carboxylesterase 5 (AtCXE5) and Carboxylesterase 12 (AtCXE12) as candidate (*Z*)-3-hexenyl acetate esterases, and assays with 2 independent T-DNA insertion mutants confirmed the involvement of AtCXE12 in the hydrolysis of (*Z*)-3-hexenyl acetate in Arabidopsis leaves. Finally, we discovered that leaf extracts from several other plant species are capable of hydrolyzing (*Z*)-3-hexenyl acetate to (*Z*)-3-hexenol, suggesting that this previously uncharacterized activity is widespread among plants.

## Results

### Arabidopsis plants take up and hydrolyze exogenous (*Z*)-3-hexenyl acetate

**Figure 1.**
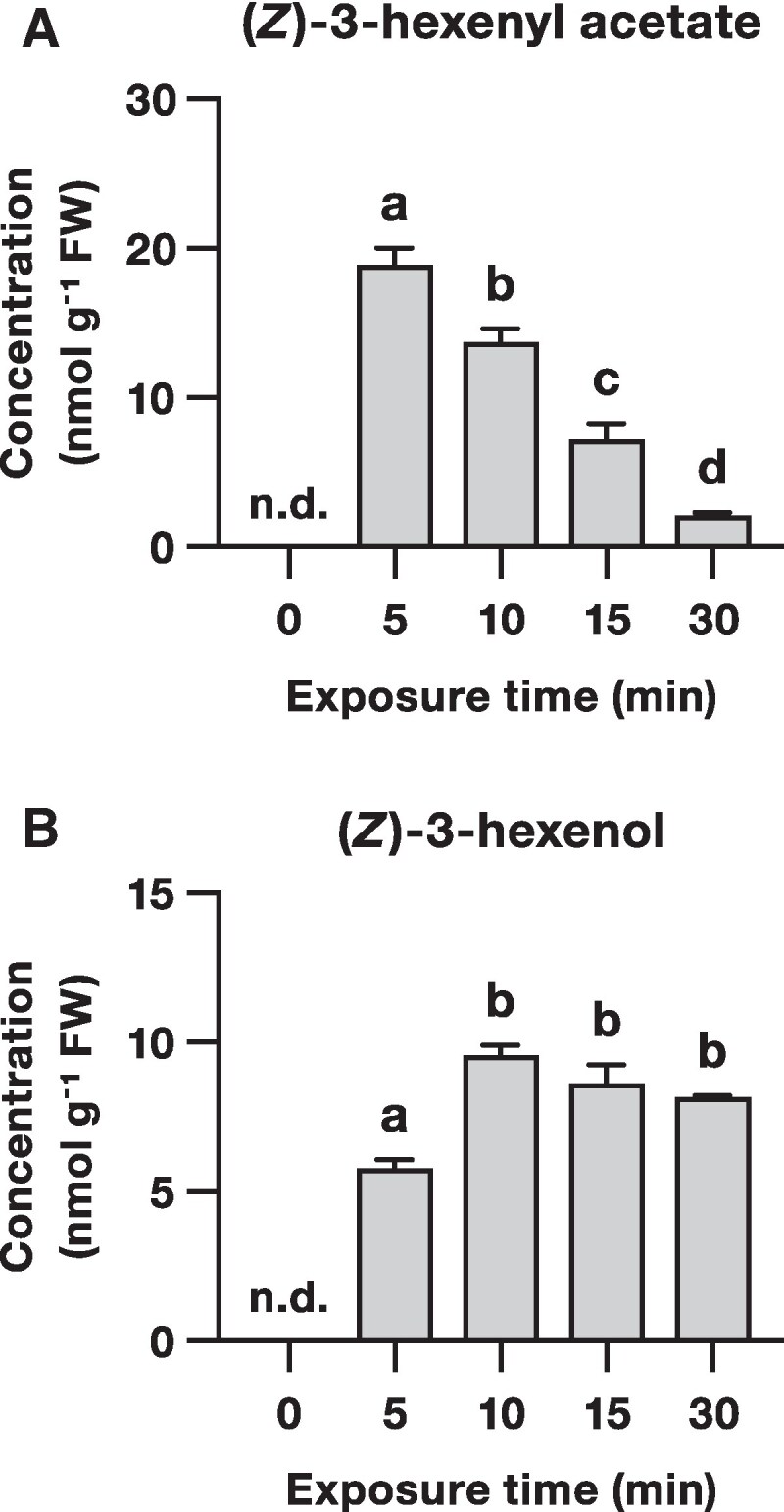
(*Z*)-3-hexenyl acetate is taken up by Arabidopsis plants and converted to (*Z*)-3-hexenol. Mean (±SEM) concentrations of **A)** (*Z*)-3-hexenyl acetate and **B)** (*Z*)-3-hexenol in leaves of Arabidopsis plants exposed to synthetic (*Z*)-3-hexenyl acetate (*n* = 4). Different letters indicate significant differences between different groups (1-way ANOVA followed by multiple comparisons by FDR-corrected LSMeans, *P* < 0.05). n.d., not detected; FW, fresh weight.

Since plants exposed to (*Z*)-3-hexenyl acetate accumulate (*Z*)-3-hexenyl glycosides (Ameye et al. 2020; Sugimoto et al. 2021), we hypothesized that exogenous (*Z*)-3-hexenyl acetate is taken up by plant tissues and hydrolyzed to (*Z*)-3-hexenol prior to glycosylation. To test our hypothesis, we exposed Arabidopsis Col-0 plants, which carry a natural mutation in the *hydroperoxide lyase* gene and are unable to produce GLVs (Duan et al. 2005), to synthetic (*Z*)-3-hexenyl acetate and measured the levels of (*Z*)-3-hexenyl acetate and (*Z*)-3-hexenol in leaves at different time points after exposure (Fig. 1). No endogenous (*Z*)-3-hexenyl acetate and (*Z*)-3-hexenol were detected in untreated leaves. Both (*Z*)-3-hexenyl acetate and (*Z*)-3-hexenol accumulated in leaves within 5 min after exposure to (*Z*)-3-hexenyl acetate. (*Z*)-3-hexenyl acetate levels decreased between 5 and 30 min after exposure, whereas (*Z*)-3-hexenol levels increased between 5 and 10 min after exposure and remained stable between 10 and 30 min. These results demonstrate that Arabidopsis plants rapidly take up exogenous (*Z*)-3-hexenyl acetate and hydrolyze it to (*Z*)-3-hexenol, suggesting the existence of a previously unidentified (*Z*)-3-hexenyl acetate esterase activity in Arabidopsis leaves.

### CXEs contribute to the (*Z*)-3-hexenyl acetate esterase activity in Arabidopsis leaves

**Figure 2.**
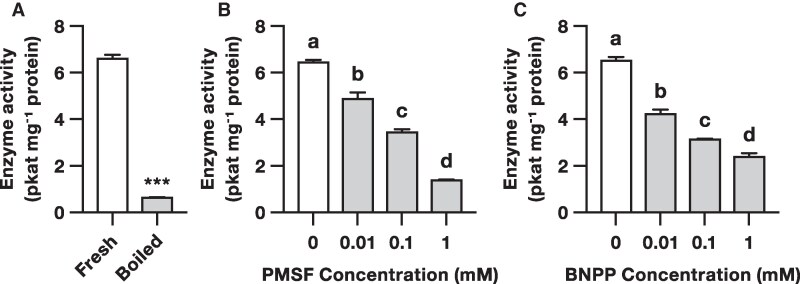
Carboxylesterases contribute to (*Z*)-3-hexenyl acetate esterase activity in Arabidopsis leaves. **A)** Mean (±SEM) (*Z*)-3-hexenyl acetate esterase activity of crude leaf extracts before and after boiling. Leaf extracts were incubated with (*Z*)-3-hexenyl acetate, and the production of (*Z*)-3-hexenol was quantified by gas chromatography-mass spectrometry (*n* = 3; representative chromatograms and mass spectra are shown in Supplementary Fig. S1). Asterisks indicate a significant difference between treatments (Student's *t*-test, ***P < 0.001). Mean (±SEM) (*Z*)-3-hexenyl acetate esterase activity of leaf extracts treated with **B)** phenylmethylsulfonyl fluoride (PMSF) or **C)** bis(*p*-nitrophenyl) phosphate (BNPP; *n* = 3). Different letters indicate significant differences between different groups (1-way ANOVA with FDR-corrected LSMeans for multiple comparisons, *P* < 0.05).

CXEs are a class of serine esterases known to hydrolyze endogenous volatile esters in fruits such as tomato (*Solanum lycopersicum*; Goulet et al. 2012) and peach (*Prunus persica*; Cao et al. 2019). To determine whether CXEs contribute to the hydrolysis of (*Z*)-3-hexenyl acetate in Arabidopsis leaves, we first assayed crude leaf extracts for (*Z*)-3-hexenyl acetate esterase activity. We found that leaf extracts hydrolyzed (*Z*)-3-hexenyl acetate to (*Z*)-3-hexenol. This activity was greatly reduced when assays were performed with denatured, heat-treated extracts (Fig. 2; Supplementary Fig. S1).

To determine whether CXEs specifically contribute to the hydrolysis of (*Z*)-3-hexenyl acetate in Arabidopsis leaves, we next tested the effects of the generic serine esterase inhibitor phenylmethylsulfonyl fluoride (PMSF) and the specific CXE inhibitor bis(*p*-nitrophenyl) phosphate (BNPP) on leaf extracts. We found that both PMSF and BNPP reduced the (*Z*)-3-hexenyl acetate esterase activity of leaf extracts in a dose-dependent manner (Fig. 2). Mean (*Z*)-3-hexenyl acetate esterase activity was reduced by 79% in the presence of 1 mm PMSF, and by 63% in the presence of 1 mm BNPP. These results indicate that serine esterases in general, and CXEs in particular, contribute significantly to (*Z*)-3-hexenyl acetate esterase activity in Arabidopsis leaves.

### AtCXE5 and AtCXE12 are candidate (*Z*)-3-hexenyl acetate esterases

The Arabidopsis genome encodes 20 carboxylesterase genes, 18 of which are expressed in leaves (Marshall et al. 2003). To determine which CXEs contribute to (*Z*)-3-hexenyl acetate hydrolysis in Arabidopsis leaves, we partially purified (*Z*)-3-hexenyl acetate esterase activity from crude leaf extracts using a 3-step purification procedure (Table 1). Leaf extracts were first fractionated by ammonium sulfate precipitation, and the fraction precipitating between 40% and 80% ammonium sulfate saturation was further purified on a phenyl-Sepharose column.

**Table 1.** Purification of (Z)-3-hexenyl acetate esterase activity from Arabidopsis leaves

Purification step	Protein	Total activity	Specific activity	Purification	Yield
	mg	pkat	pkat mg⁻¹	fold	%
Crude extract	364	1848	5.08	1.00	100
Ammonium sulfate	102	1079	10.5	2.08	58
Phenyl Sepharose	2.03	683	336	66.3	37
Mono Q	0.23	194	838	165	10

The elution profile of the phenyl-Sepharose column indicated that (*Z*)-3-hexenyl acetate esterase activity was distributed into 3 distinct pools of unequal size (Supplementary Fig. S2). The largest pool, comprising approximately 80% of the total esterase activity recovered at this step, was further resolved into a single activity peak on a Mono Q column.

Proteomic analysis of the 3 most active fractions collected from the Mono Q column identified 85 Arabidopsis proteins with at least 2 peptides, including 2 putative CXEs: AtCXE5 (At1g49660) and AtCXE12 (At3g48690; Supplementary Table S1; Supplementary Fig. S3).

### Recombinant AtCXE5 and AtCXE12 hydrolyze (*Z*)*-*3-hexenyl acetate

The full-length coding sequences of AtCXE5 and AtCXE12 were heterologously expressed in Escherichia coli, and the recombinant proteins were tested for esterase activity with (*Z*)-3-hexenyl acetate and several other (*Z*)-3-hexenyl esters (Table 2). Both AtCXE5 and AtCXE12 showed relatively high activity with (*Z*)-3-hexenyl acetate, (*Z*)-3-hexenyl propionate, and (*Z*)-3-hexenyl butyrate, but little or no activity with (*Z*)-3-hexenyl isobutyrate, (*Z*)-3-hexenyl 2-methylbutyrate, and (*Z*)-3-hexenyl tiglate. The optimum pH for both proteins was found to be 7.5 in Tris-HCl buffer.

**Table 2.** Substrate specificity of AtCXE5 and AtCXE12

Substrate	Relative activity, %^a^	
	AtCXE5	AtCXE12
(*Z*)-3-hexenyl acetate	100.00 ± 8.88	100.00 ± 14.17
(*Z*)-3-hexenyl propionate	57.59 ± 8.37	180.72 ± 15.54
(*Z*)-3-hexenyl butyrate	101.78 ± 4.65	299.33 ± 5.36
(*Z*)-3-hexenyl isobutyrate	5.29 ± 0.59	6.65 ± 1.13
(*Z*)-3-hexenyl 2-methylbutyrate	n.d.	6.21 ± 1.15
(*Z*)-3-hexenyl tiglate	n.d.	n.d.

^a^All assays were performed under identical reaction conditions. Activates are presented as a percentage relative to (*Z*)-3-hexenyl acetate (100%). Data are mean ± SEM (*n* = 3). n.d., nondetectable activity (<2%).

Kinetic analyses revealed that both AtCXE5 and AtCXE12 preferred (*Z*)-3-hexenyl butyrate as substrate over (*Z*)-3-hexenyl propionate and (*Z*)-3-hexenyl acetate (Table 3). The apparent *K*ₘ value of AtCXE5 for (*Z*)-3-hexenyl acetate was 1.45 times higher than that of AtCXE12, while the *k*_cat_ values for (*Z*)-3-hexenyl acetate were comparable for both enzymes. Overall, the catalytic efficiency (*k*_cat_/*K*ₘ) of AtCXE5 for (*Z*)-3-hexenyl acetate was 1.68 times lower than that of AtCXE12.

**Table 3.** Kinetic parameters of AtCXE5 and AtCXE12

Enzyme	Substrate	Kₘ	Vmax	k_cat_	k_cat_/Kₘ
		mM	µM min⁻¹	s⁻¹	mM⁻¹ s⁻¹
AtCXE5	(*Z*)-3-hexenyl acetate	8.63 ± 1.28	108.14 ± 8.19	3.17	0.38
	(*Z*)-3-hexenyl propionate	1.45 ± 0.19	10.25 ± 0.36	0.30	0.21
	(*Z*)-3-hexenyl butyrate	0.24 ± 0.03	5.36 ± 0.14	0.16	0.65
AtCXE12	(*Z*)-3-hexenyl acetate	5.94 ± 0.84	127.39 ± 7.48	3.78	0.64
	(*Z*)-3-hexenyl propionate	2.26 ± 0.34	75.93 ± 3.41	2.25	0.99
	(*Z*)-3-hexenyl butyrate	0.46 ± 0.05	27.54 ± 0.68	0.85	1.86

Data are mean ± SEM (*n* = 3).

### *AtCXE12* contributes to the hydrolysis of exogenous (*Z*)-3-hexenyl acetate in Arabidopsis leaves

**Figure 3.**
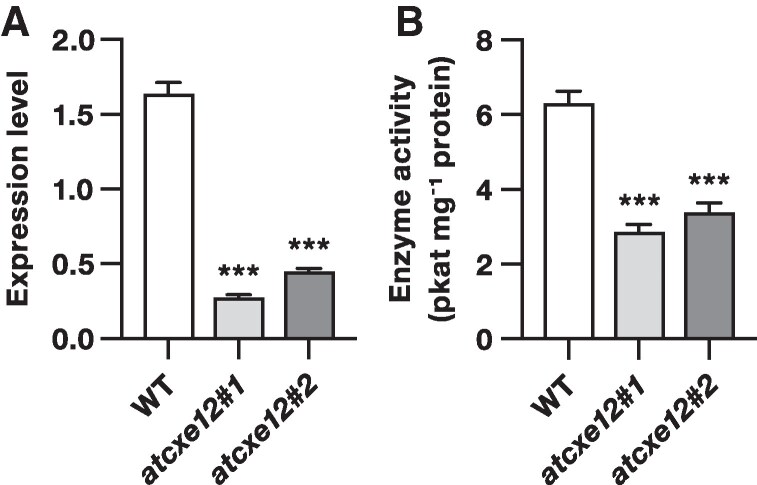
(*Z*)-3-hexenyl acetate esterase activity is reduced in leaf extracts of atcxe12 mutants compared with WT plants. **A)** Mean (± SEM) expression levels of *AtCXE12* in *atcxe12* mutants and wild type (WT) plants (*n* = 5). Asterisks indicate significant differences between the WT and *atcxe12* mutants (Student's *t*-test; ***P < 0.001). **B)** Mean (± SEM) (*Z*)-3-hexenyl acetate esterase activity of crude leaf extracts from *atcxe12* mutants and WT plants (*n* = 5). Asterisks indicate significant differences between the WT and *atcxe12* mutants (Student's *t*-test; ***P < 0.001).

A search of the T-DNA Express database identified 2 T-DNA insertion alleles of *AtCXE12*: one previously characterized by Gershater et al. (2007) with a 5′UTR insertion (*atcxe12#1*) and a second uncharacterized allele with a 3′UTR insertion (*atcxe12#2*). RT-qPCR analysis revealed that *AtCXE12* transcripts were reduced by 84% in the *atcxe12#1* mutant and by 73% in the *atcxe12#2* mutant compared with WT. Correspondingly, (*Z*)-3-hexenyl acetate esterase activity of leaf extracts was reduced by 55% in the *atcxe12#1* mutant and by 47% in the *atcxe12#2* mutant compared with the WT (Fig. 3).

Similarly, 2 T-DNA insertion alleles of *AtCXE5* were identified, both with insertions in the 5′ UTR (designated *atcxe5#1* and *atcxe5#2*). *AtCXE5* transcript levels in the *atcxe5#1 mutant* were comparable to those in WT plants, whereas the *atcxe5#2* mutant showed a 27% reduction in transcript levels. No significant difference in esterase activity was observed between either *atcxe5* mutant and WT plants (Supplementary Fig. S4); therefore, we focused solely on AtCXE12 for further investigation.

**Figure 4.**
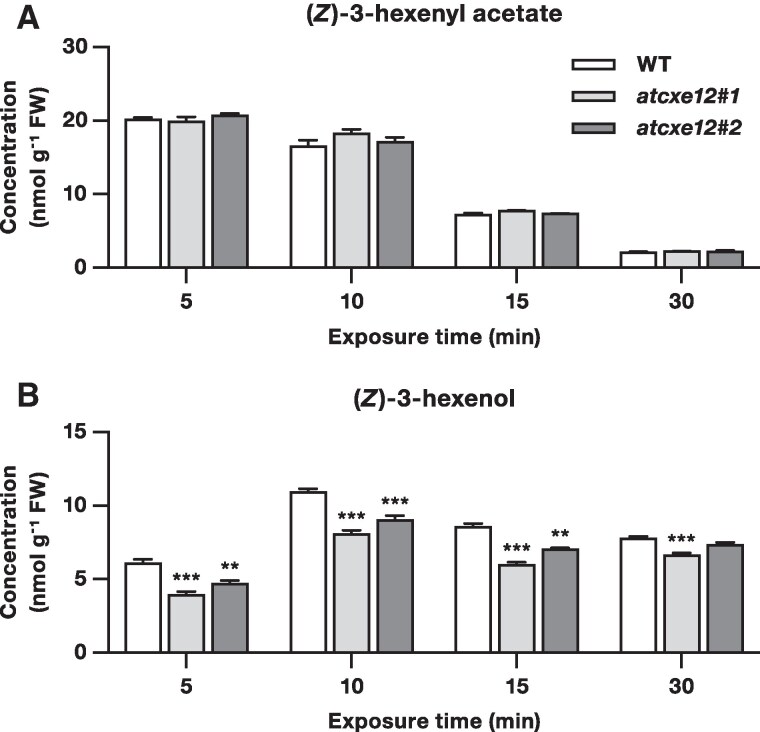
AtCXE12 converts (*Z*)-3-hexenyl acetate to (*Z*)-3-hexenol in Arabidopsis leaves. Mean (± SEM) concentrations of **A)** (*Z*)-3-hexenyl acetate and **B)** (*Z*)-3-hexenol in leaves of *atcxe12* mutants and wild type (WT) plants exposed to synthetic (*Z*)-3-hexenyl acetate (*n* = 6). Asterisks indicate significant differences between the WT and *atcxe12* mutants (Student's *t*-test; **P < 0.01, ***P < 0.001), while the absence of asterisks indicates no significant differences. FW, fresh weight.

To investigate the contribution of AtCXE12 to the hydrolysis of exogenous (*Z*)-3-hexenyl acetate in Arabidopsis leaves, we measured the levels of (*Z*)-3-hexenyl acetate and (*Z*)-3-hexenol in leaves of *atcxe12* mutants and WT plants after exposure to synthetic (*Z*)-3-hexenyl acetate. However, (*Z*)-3-hexenol levels were consistently lower in leaves of both *atcxe12* mutants compared with WT plants after exposure to (*Z*)-3-hexenyl acetate (Fig. 4). The *atcxe12#1* mutant showed an approximate 29% reduction in (*Z*)-3-hexenol levels relative to WT at all time points, while the *atcxe12#2* mutant showed a similar, albeit smaller, 16% reduction in (*Z*)-3-hexenol levels.

### (*Z*)-3-hexenyl acetate esterase activity is conserved across different plant species

**Figure 5.**
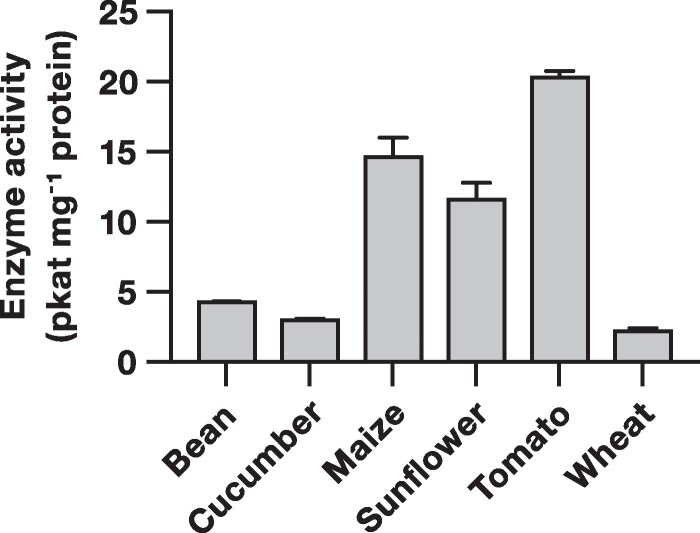
(*Z*)-3-hexenyl acetate esterases are present in leaf extracts from different plant species. Mean (±SEM) (*Z*)-3-hexenyl acetate esterase activity of crude leaf extracts from different plant species. Leaf extracts were incubated with (*Z*)-3-hexenyl acetate and the production of (*Z*)-3-hexenol was quantified by gas chromatography-mass spectrometry (*n* = 3).

Finally, we tested crude leaf extracts from different plant species to investigate how widely conserved (*Z*)-3-hexenyl acetate esterases are. We found that leaf extracts from bean (*Phaseolus vulgaris*), cucumber (*Cucumis sativus*), maize (*Zea mays* subsp. *mays*), sunflower (*Helianthus annuus*), tomato, and wheat (*Triticum aestivum*) all hydrolyze (*Z*)-3-hexenyl acetate to (*Z*)-3-hexenol (Fig. 5). (*Z*)-3-hexenyl acetate esterase activity was relatively high in leaf extracts from tomato, maize, and sunflower, whereas leaf extracts from bean, cucumber, and wheat showed lower activity. Thus, (*Z*)-3-hexenyl acetate esterase activity is likely to be conserved throughout the plant kingdom, although the ability to hydrolyze (*Z*)-3-hexenyl acetate is expected to vary between species.

## Discussion

The differential metabolism of GLVs in plant tissues can modulate the specific activities of these compounds (Shiojiri et al. 2006; Matsui et al. 2012; Matsui 2016; Sugimoto et al. 2021). Accordingly, characterizing the enzymes involved in GLV metabolism is crucial for understanding their potential functions. In this study, we demonstrate that (*Z*)-3-hexenyl acetate is rapidly converted to (*Z*)-3-hexenol in Arabidopsis leaves and identify 2 CXEs that catalyze this reaction.

CXEs are known to hydrolyze volatile esters in fruits of several domesticated plants. For example, SlCXE1 from tomato is expressed primarily in fruit tissues and can hydrolyze (*Z*)-3-hexenyl acetate and other volatile esters that contribute to fruit flavor (Goulet et al. 2012). Similarly, PpCXE1 from peach (Cao et al. 2019) and FanCXE1 from strawberry (*Fragaria* × *ananassa*; Martínez-Rivas et al. 2022) are involved in the hydrolysis of volatile esters during fruit ripening. Our results show that CXEs also play an important role in the hydrolysis of (*Z*)-3-hexenyl acetate in Arabidopsis leaves. In particular, we found that AtCXE5 and AtCXE12 both hydrolyze (*Z*)-3-hexenyl acetate in vitro and exhibit high relative activity with longer, straight-chain (*Z*)-3-hexenyl esters. While the kinetic parameters of AtCXE5 and AtCXE12 are comparable to other known CXEs (http://brenda-enzymes.info), their apparent *K*ₘ values for (*Z*)-3-hexenyl acetate are higher than those reported for SlCXE1 (*K*ₘ = 0.47) and PpCXE1 (*K*ₘ = 0.99). Several factors could explain these differences. For example, AtCXE5 and AtCXE12 may be adapted to function efficiently at relatively high (*Z*)-3-hexenyl acetate concentrations, consistent with the levels of (*Z*)-3-hexenyl acetate found near wounded leaves (D'Auria et al. 2007). In addition, consumer preferences during domestication may have led to the selection of alleles associated with lower levels of (*Z*)-3-hexenyl acetate in ripening fruits, driving the evolution of CXEs in domesticated fruits toward a higher affinity for (*Z*)-3-hexenyl acetate and other volatile esters with undesirable off-flavors.

While some CXEs exhibit high substrate specificity, others are more promiscuous, acting on a wide range of endogenous and exogenous esters (Marshall et al. 2003). AtCXE5 and AtCXE12 fall into the latter category, hydrolyzing (*Z*)-3-hexenyl acetate as well as volatile esters not typically produced by Arabidopsis, such as (*Z*)-3-hexenyl propionate and (*Z*)-3-hexenyl butyrate. Notably, both enzymes have also been shown to hydrolyze the model ester *p*-nitrophenyl acetate, while AtCXE12 alone can convert methyl 2,4-dichlorophenoxyacetate to the active herbicide 2,4-dichlorophenoxyacetic acid (Gershater et al. 2007).These results suggest that AtCXE5 and AtCXE12 can hydrolyze a number of different esters in Arabidopsis leaves, although it remains to be determined whether either enzyme has a clear substrate preference.

What is the biological significance of converting (*Z*)-3-hexenyl acetate to (*Z*)-3-hexenol in plant leaves? One possibility is that hydrolysis of (*Z*)-3-hexenyl acetate prevents it from binding to an unidentified receptor, thereby downregulating (*Z*)-3-hexenyl acetate-induced defense responses. Alternatively, hydrolysis may serve to activate (*Z*)-3-hexenyl acetate by converting it to (*Z*)-3-hexenol, which subsequently stimulates plant defenses. This second possibility is similar to the process by which plants convert the volatiles methyl salicylate and methyl jasmonate to the active phytohormones salicylic acid and jasmonic acid, respectively (Forouhar et al. 2005; Koo et al. 2013).In both cases, silencing the methylesterase responsible for their hydrolysis attenuated plant responses to these volatile signaling molecules (Park et al. 2007; Wu et al. 2008; Manosalva et al. 2010).

Our results show that *atcxe12* mutants accumulate significantly lower levels of (*Z*)-3-hexenol after exposure to (*Z*)-3-hexenyl acetate compared with WT plants. The less than 30% reduction in (*Z*)-3-hexenol accumulation observed in *atcxe12* mutants suggests that the remaining esterase activity may be attributed either to residual *AtCXE12* expression in the mutant background or to the involvement of additional (*Z*)-3-hexenyl acetate esterases that have yet to be identified in Arabidopsis. This latter possibility is supported by our initial purification results (Supplementary Fig. S2), which revealed multiple distinct activity peaks in the elution profile, strongly indicating that several enzymes contribute to (*Z*)-3-hexenyl acetate hydrolysis in Arabidopsis leaves.

A promising candidate is *AtCXE5*, which co-purified with *AtCXE12* and demonstrated the ability to hydrolyze (*Z*)-3-hexenyl acetate in vitro. To investigate the role of *AtCXE5* in the hydrolysis of exogenous (*Z*)-3-hexenyl acetate in Arabidopsis, future experiments could include generating *atcxe5* single mutants and *atcxe5*/*atcxe12* double mutants to assess potential additive effects on reducing (*Z*)-3-hexenyl acetate levels in leaves. Additionally, overexpressing *AtCXE5* in the *atcxe12* background could help determine whether increased *AtCXE5* activity compensates for the loss of *AtCXE12*.

Despite showing reduced esterase activity, atcxe12 mutants did not accumulate higher levels of exogenous (*Z*)-3-hexenyl acetate. This may be due to the continuous uptake of (*Z*)-3-hexenyl acetate from the surrounding headspace, which could replenish (*Z*)-3-hexenyl acetate levels in leaves faster than they can be hydrolyzed. Alternatively, the (Z)-3-hexenol produced from hydrolysis could be re-esterified back to (*Z*)-3-hexenyl acetate (D'Auria et al. 2007), contributing to its accumulation in the leaves. Further studies, including the development of higher-order GLV mutants and overexpression lines, will be critical for providing the molecular tools needed to clarify why plants convert (*Z*)-3-hexenyl acetate to (*Z*)-3-hexenol. Applying the knowledge gained here to plants that exhibit a strong defense response to (*Z*)-3-hexenyl acetate will be essential in this context.

## Materials and Methods

### Plant material and growth conditions

Arabidopsis (*A. thaliana* (L.) Heynh.) ecotype Columbia (Col-0) was used as the wild type (WT) for all experiments. The T-DNA insertion mutants *atcxe5#1* (SALK_209596C), *atcxe5#2* (SALK_106155C1), *atcxe12#1* (SAIL_445_G03), and *atcxe12#2* (SALK_058614C) were obtained from the Arabidopsis Biological Resource Center (Ohio State University, Columbus, OH, USA). *atcxe12#1* has been described previously (Gershater et al. 2007). All T-DNA insertion mutants were genotyped using a T-DNA-specific primer and gene-specific primer pairs flanking each insertion site (Supplementary Table S2).

Arabidopsis plants were grown on a mixture of PRO-MIX FLX (Premier Horticulture Inc., Quakertown, PA, USA) and perlite (3:1, v/v), supplemented with Osmocote Smart Release 14-14-14 plant food (Scotts Company LLC, Marysville, OH, USA) in a growth chamber (22 °C) under a 10:14, light/dark cycle with illumination from cool-white fluorescent lamps (∼120 *µ*mol photons m⁻² s⁻¹). All other plants (Supplementary Table S3) were grown on commercial potting soil (Selmaterra, Bigler Samen AG, Steffisburg, CH) in a climate-controlled greenhouse (26 °C) under a 16:8, light/dark cycle, with supplemental lighting from metal halide and high-pressure sodium lamps (400 W).

### (*Z*)-3-hexenyl acetate exposure treatments

To expose Arabidopsis plants to (*Z*)-3-hexenyl acetate, individual 4- to 5-wk-old plants were placed in a 500-cm³ glass container together with a cotton swab treated with 0.5 *µ*mol of synthetic (*Z*)-3-hexenyl acetate dissolved in 5 *µ*L of dichloromethane. Whole rosettes were harvested at various times after exposure and snap frozen in liquid nitrogen. Samples were ground in liquid nitrogen and stored at −80 °C until use.

### Quantification of internal (*Z*)-3-hexenyl acetate and (*Z*)-3-hexenol

Internal (*Z*)-3-hexenyl acetate and (*Z*)-3-hexenol were extracted from Arabidopsis leaves according to a protocol modified from Seidl-Adams et al. (2015). Briefly, 200 mg of ground plant material was homogenized in 0.8 mL *n*-hexane using a FastPrep F120 tissue homogenizer (Qbiogene, Carlsbad, CA, USA). The homogenate was centrifuged at 15,000 × *g* for 3 min, and the upper organic phase was transferred to a 4 mL glass vial. Volatiles were collected by vapor phase extraction on a SuperQ filter trap (20 mg; Alltech Associates, Deerfield, IL, USA) as described in Schmelz et al. (2004). Trapped volatiles were eluted with 100 *µ*L *n*-hexane/dichloromethane (1:1, v/v) containing 500 ng nonyl acetate as internal standard.

Samples were analyzed on an Agilent 6890 series gas chromatograph (GC) coupled to an Agilent 5973 quadrupole mass selective detector (MS; interface temperature, 250 °C; quadrupole temperature, 150 °C; source temperature, 230 °C; electron energy, 70 eV). An aliquot of each sample was injected in splitless mode onto an HP-5MS column (30 m × 0.25 mm i.d. × 0.25 *µ*m thickness; Agilent, Palo Alto, CA, USA). Helium was used as the carrier gas at a constant flow rate of 1 mL min⁻¹. The oven temperature was held at 40 °C for 2 min, then increased to 280 °C at 15 °C min⁻¹ and held at 280 °C for 3 min. Mass spectra were acquired in scan mode from 50 to 300 *m/z*. Absolute quantities were determined using a standard curve.

### Preparation of crude leaf extract and esterase activity assays

Ground leaf tissue was homogenized in 10 volumes (w/v) of ice-cold extraction buffer (50 mm Tris-HCl, pH 7.5, 10% glycerol (w/v), 2 mm dithiothreitol). The homogenate was centrifuged at 15,000 × *g* for 10 min, and the supernatant was collected for esterase activity assays. Total protein concentration was determined using the Bradford method (Bradford 1976) using bovine serum albumin as a standard.

Esterase activity was quantified by measuring the amount of (*Z*)-3-hexenol produced from the hydrolysis of (*Z*)-3-hexenyl esters. Standard assays were performed in a 200-*µ*L reaction mixture containing 50 mm of Tris-HCl buffer (pH 7.5), 0.2 mm of volatile ester substrate, and varying amounts of protein solution. Reaction mixtures were incubated at 30 °C for 20 min, and then extracted with 100 *µ*L of *n*-hexane/dichloromethane (1:1, v/v) containing 500 ng of nonyl acetate as an internal standard. Extracts were analyzed by GC-MS as previously described.

To evaluate the effects of the generic serine esterase inhibitor PMSF and the specific carboxylesterase inhibitor bis(*p*-nitrophenyl) phosphate (BNPP) on esterase activity, leaf extracts were incubated with varying concentrations of each inhibitor for 1 h at 23 °C. Inhibitor-treated extracts were then assayed for esterase activity under standard assay conditions.

### Esterase purification and proteomic analysis

Approximately 100 g of ground leaf tissue was homogenized in 150 mL of ice-cold extraction buffer (50 mm Tris-HCl, pH 7.5, 2 mm dithiothreitol). The homogenate was filtered through 3 layers of muslin to remove debris, and the filtrate was centrifuged at 10,000 × *g* for 20 min to obtain a crude extract as the supernatant.

The crude extract was fractionated with ammonium sulfate between 40% and 80% saturation, and the precipitate was dissolved in buffer A (50 mm Tris-HCl, pH 7.5, 0.5 m ammonium sulfate). The protein solution was applied to a 5-mL phenyl Sepharose HP column (Cytiva, Marlborough, MA, USA) equilibrated with buffer A. The column was washed with 25 mL of buffer A, and bound proteins were eluted over an 80-mL decreasing linear gradient to 0 mm ammonium sulfate at a flow rate of 1 mL min⁻¹. Fractions were collected at 2-mL intervals and assayed for (*Z*)-3-hexenyl acetate esterase activity.

Active fractions were pooled and exchanged into buffer B (50 mm Tris-HCl, pH 7.5) before being applied to a Mono Q HR 5/5 column (GE Healthcare, Piscataway, NJ, USA) equilibrated with buffer B. The column was washed with 15 mL of buffer B, and bound proteins were eluted over a 30-mL increasing linear gradient to 0.2 m NaCl at a flow rate of 0.5 mL min⁻¹. Fractions were collected at 0.5-mL intervals and assayed for (*Z*)-3-hexenyl acetate esterase activity. Active fractions were pooled for proteomic analysis.

Prior to proteomic analysis, proteins were alkylated with iodoacetamide and digested with Trypsin/Lys-C Mix (Promega, Madison, WI, USA), according to the manufacturer's instructions. Digested peptides were analyzed on a Bruker nanoElute liquid chromatography system coupled to a Bruker timsTOF fleX mass spectrometer operated in PASEF mode with a capillary voltage of 1.6 kV. Peptides were separated on a reversed-phase C18 column (15 cm × 0.75 *µ*m i.d., 1.7 *µ*m particle size, 100 Å pore size; PepSep, Marslev, DK) maintained at 50 °C. Water/formic acid (100:0.1, v/v) and acetonitrile/formic acid (100:0.1, v/v) were used as mobile phases A and B, respectively. The elution profile was as follows: 0 to 17.22 min, 5% to 30% A in B; 17.22 to 17.72 min, 30% to 95% A in B; 17.72 to 20.85, 95% A in B; and 20.85 to 30 min 5% A in B, with a flow rate of 0.3 *µ*L min⁻¹.Mass spectra were acquired from 100 to 1,700 *m/z*. Data were analyzed using Byonic software (v3.6.0; Protein Metrics Inc., Cupertino, CA, USA) against the NCBI RefSeq Arabidopsis database and a list of 536 common laboratory contaminants.

### RNA extraction and cDNA synthesis

Total RNA was extracted from ground leaf tissue using the RNeasy Mini Plant Kit (Qiagen, Valencia, CA, USA) and treated with TURBO DNase using the TURBO DNA-free Kit (Applied Biosystems, Carlsbad, CA, USA) according to the manufacturer's instructions. First-strand cDNA was synthesized from 1 to 2 *µ*g of DNase-treated total RNA using SuperScript IV Reverse Transcriptase (Invitrogen, Carlsbad, CA, USA) and a mixture of anchored oligo(dT)18 and random octamer primers (Genomics Core Facility, The Pennsylvania State University, University Park, PA, USA).

### Cloning and heterologous expression

Full-length coding sequences were amplified from cDNA using gene-specific primers (Supplementary Table S2) and ligated into the pET28b expression vector (Novagen, Madison, WI, USA), in-frame with an N-terminal hexahistidine (His)-tag. Constructs were transformed into Escherichia coli Rosetta 2 (DE3)pLysS cells (Novagen) for heterologous expression. Cells harboring an expression construct were grown at 37 °C in Lysogeny Broth supplemented with kanamycin (50 *µ*g mL⁻¹) and chloramphenicol (50 *µ*g mL⁻¹) to an OD₆₀₀ of 0.7 to 0.8. Cultures were cooled to 23 °C, and isopropyl-β-D-1-thiogalactopyranoside was added to a final concentration of 1 mm. Induced cultures were incubated at 16 °C with continuous shaking (200 rpm) for 20 h, after which cells were harvested by centrifugation and stored at −80 °C until use.

### Enzyme kinetics

Protein concentration and incubation time were chosen so that the reaction velocity was linear over the incubation time period. The pH optimum was determined in 50 mm of citrate buffer (pH 4.0 to 6.0), 50 mm of sodium phosphate buffer (pH 6.0 to 7.5), or 50 mm of Tris-HCl buffer (pH 7.5 to 9.0). Standard assays were performed in a 200-µL reaction mixture containing 50 mm of Tris-HCl buffer (pH 7.5), 5 to 16,000 *µ*M of substrate, and 0.5 *µ*M (∼4 *µ*g) of affinity-purified recombinant protein. Reaction mixtures were incubated at 30 °C for 10 min, and then extracted with 100 *µ*L of *n*-hexane/dichloromethane (1:1, v/v) containing 500 ng of nonyl acetate as an internal standard. Extracts were analyzed by GC-MS as previously described. All assays were performed in triplicate, and data were fitted to the Michaelis–Menten equation by non-linear regression.

### Gene expression analysis

RT-qPCR was performed using ORA SEE qPCR Mix (highQu GmbH, Germany) on an Applied Biosystems QuantStudio 5 Real-Time PCR system. A linear standard curve was constructed using a serial dilution of cDNA pooled from each sample, and was generated by plotting the log₁₀ of the dilution factors against the threshold cycle (Ct). Relative transcript levels of the target genes were quantified according to the standard curve. The *AtPP2AA3* gene (At1g13320) was used as an internal standard to normalize cDNA concentrations. Primers used in this study are listed in Supplementary Table S2.

### Accession Numbers

Sequence information from this article is found in The Arabidopsis Information Resource database (www.arabidopsis.org) under accession numbers: AtCXE5 (At1g49660) and AtCXE12 (At3g48690).

## Supplementary Material

kiaf119_Supplementary_Data

## Data Availability

The data underlying this article will be shared on reasonable request to the corresponding author.

## Supplementary data

The following materials are available in the online version of this article.

**Supplementary Figure S1.** Conversion of (*Z*)-3-hexenyl acetate to (*Z*)-3-hexenol by crude Arabidopsis leaf extracts.

**Supplementary Figure S2.** Purification of (*Z*)-3-hexenyl acetate esterase activity from Arabidopsis leaves.

**Supplementary Figure S3.** Sequence coverage of AtCXE5 and AtCXE12 peptides identified by proteomic analysis.

**Supplementary Figure S4.** Characterization of *atcxe5* T-DNA insertion lines.

**Supplementary Table S1.** List of proteins identified by proteomic analysis of (*Z*)-3-hexenyl acetate esterase activity purified from Arabidopsis leaves.

**Supplementary Table S2.** Primers used in this study.

**Supplementary Table S3.** Plant material used in this study.

## Dive Curated Terms

The following phenotypic, genotypic, and functional terms are of significance to the work described in this paper:

AtCXE5 Gramene: AT1G49660
AtCXE5 Araport: AT1G49660

AtCXE12 Gramene: AT3G48690
AtCXE12 Araport: AT3G48690
