# Supplementary material for: The carboxylesterase AtCXE12 converts volatile (Z)-3-hexenyl acetate to (Z)-3-hexenol in Arabidopsis leaves
Source: Plant Physiol. 2025 Mar 27;197(4):kiaf119. doi: 10.1093/plphys/kiaf119 (PMC11986948; doi:10.1093/plphys/kiaf119)
Supplement: kiaf119_Supplementary_Data [file kiaf119_supplementary_data.pdf]

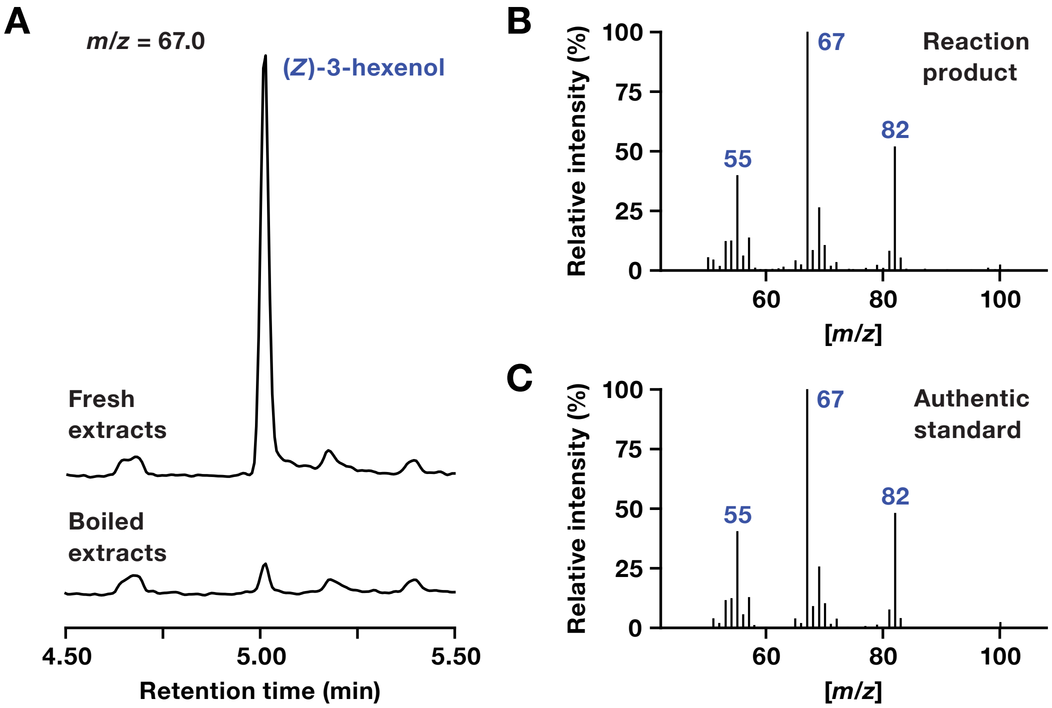

Supplementary Figure S1. Conversion of (*Z*)-3-hexenyl acetate to (*Z*)-3-hexenol by crude Arabidopsis leaf extracts. (A) Gas chromatography mass GC-MS analysis of (*Z*)-3-hexenol found in a reaction mixture containing fresh or boiled crude leaf extracts and (*Z*)-3-hexenyl acetate. Mass spectra of (B) (*Z*)-3-hexenol formed after incubation of fresh crude leaf extracts with (*Z*)-3-hexenyl acetate and (C) authentic (*Z*)-3-hexenol.

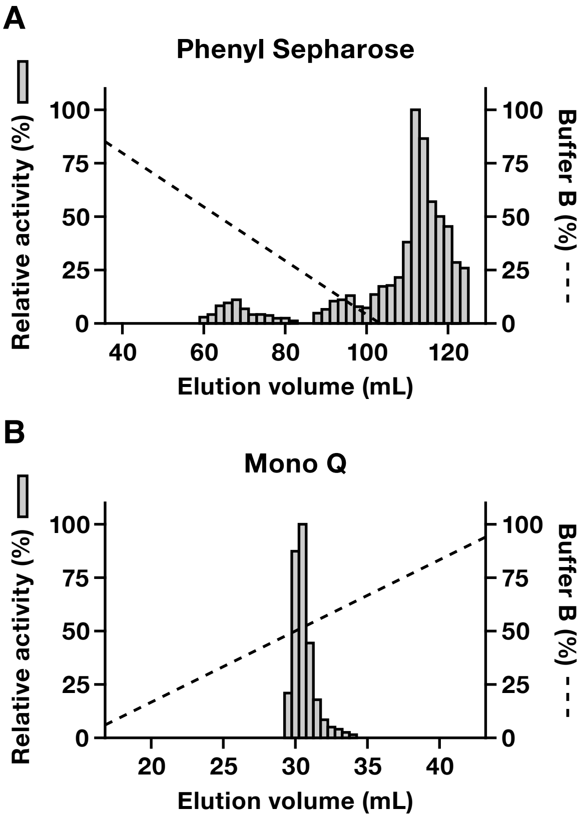

Supplementary Figure S2. Purification of (*Z*)-3-hexenyl acetate esterase activity from Arabidopsis leaves. Elution profiles of (*Z*)-3-hexenyl acetate esterase activity from (A) phenyl Sepharose and (B) Mono Q columns.

AtCXE5 (At1g49660)

MES**EIASEFL PFCRIYKDGR VERLIGTDTI PASLDPTYDV VSKDVIYSPE NNLSVRLFLP** 60

HKSTKLTAGN KLPLLIYIHG GAWIIESPFS PLYHNYLTEV VKSANCLAVS VQYRRAPEDP 120

VPAAYEDVWS AIQWIFAHSN GSGPVDWINK HADFG**KVFLG GDSAGGNISH HMAMKA**GKEK 180

KLDLKIKGIA VVHPA**FWGTD PVDEYDVQDK ETRS**GIAEIW EKIASPNSVN GTDDPLFNVN 240

GSGSDFSGLG CDKV**LVAVAG KDVFVRQGLA YAAKLEK**CEW EGTV**EVVEEE GEDHVFHLQN** 300

PKSDKALKFL KKFVEFIIG 319

AtCXE12 (At3g48690)

MDS**EIAVDCS PLLKIYKSGR IERLMGEATV PPSSEPQNGV VSKDVVYSAD NNLSVRI**YLP 60

EKAAAETDSK LPLLVYFHGG GFIIETAFSP TYHTFLTTSV SASNCVAVSV DY**RRAPEHPI** 120

SVPFDDSWTA LKWVFTHITG SGQEDWLNKH ADFS**RVFLSG DSAGANIVHH MAMRA**AKEKL 180

SPGLNDTGIS GII**LLHPYFW SKTPIDEKDT KDETLRM**KIE AFWMMASPNS KDGTDDPL**LN** 240

VVQSESVDLS GLGCGKVLVM VAEKDALVRQ GWGYAAKLEK SGWKGEVEVV ESEGEDHVFH 300

LLKPECDNAI EVMHKFSGFI KGGN 324

Supplementary Figure S3. Sequence coverage of AtCXE5 and AtCXE12 peptides identified by proteomic analysis. Deduced amino acid sequence of AtCXE5 and AtCXE12. Peptides identified by proteomic analysis are shown in bold.

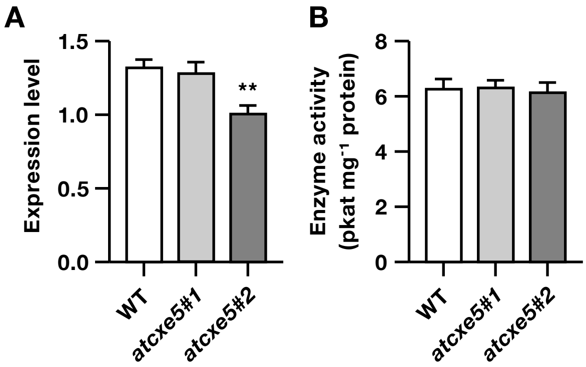

Supplementary Figure S4. Characterization of *atcxe5* T-DNA insertion lines. (A) Mean (± SEM) expression levels of *AtCXE5* in *atcxe5* mutants and wild type (WT) plants (n = 5) . Asterisks indicate a significant difference between treatments (Student's t-test, **P < 0.01). (B) Mean (± SEM) (*Z*)-3-hexenyl acetate esterase activity of crude leaf extracts from *atcxe5* mutants and WT plants (n = 5).

Supplementary Table S1. List of proteins identified by proteomic analysis of (Z)-3-hexenyl acetate esterase activity purified from Arabidopsis leaves.

Accession number	Name	Total intensity	# of spectra	# of unique peptides	Coverage, %
NP_567103	Transketolase	5949487.7	151	59	53.31
NP_187593	Adenosine kinase 1	5365525.9	130	52	59.88
NP_194098	Polyketide cyclase/dehydrase and lipid transport superfamily protein	9021641.8	154	42	84.77
NP_190861	Aldolase superfamily protein	1627294.6	58	31	65.08
NP_200333	Trigger factor type chaperone family protein	1280158.9	50	30	31.63
NP_177875	PDI-like 1-2	1472352.2	37	23	39.96
NP_568203	ATP synthase alpha/beta family protein	1258030.6	42	19	30.4
NP_566796	Glyceraldehyde 3-phosphate dehydrogenase A subunit	1612348.7	51	25	30.3
NP_190438	alpha/beta-Hydrolases superfamily protein (CXE12)	1340105.8	45	22	58.02
NP_001321033	Ribosomal protein S5/Elongation factor G/III/V family protein	1033774.6	38	23	19.69
NP_001326777	Carbonic anhydrase 1	1087006.7	42	17	33.98
NP_001189586	Putative BCR, YbaB family COG0718	1029915.4	27	12	48.33
NP_001323480	Glutathione S-transferase phi 8	918498.8	41	18	47.91
NP_195950	Adenosine kinase 2	725480.9	29	15	30.72
NP_192161	Glutathione S-transferase phi 2	770360.1	28	16	51.41
NP_001189959	Dehydratase family	779707.8	28	15	20.79
NP_187884	Phosphoglycerate kinase 1	478440.1	20	12	27.65

NP_175389	Carboxylesterase 5 (CXE5)	659779.3	22	12	43.57
NP_001030991	Ascorbate peroxidase 1	596821.2	17	8	33.6
NP_192705	Oxidoreductase family protein	744839.5	27	12	26.24
NP_567934	Pyridoxal phosphate (PLP)-dependent transferases superfamily protein	361318.7	11	7	20.39
NP_001330235	Carbonic anhydrase 2	393149.3	12	7	23.17
NP_001031721	Aldolase superfamily protein	404309.2	11	7	29.33
NP_174996	Glyceraldehyde-3-phosphate dehydrogenase B subunit	508541.9	22	8	11.86
NP_194688	S-adenosyl-L-methionine-dependent methyltransferases superfamily protein	191745.4	6	6	20.82
NP_174499	Phospholipid/glycerol acyltransferase family protein	394318.4	16	8	19.83
NP_197423	ADP glucose pyrophosphorylase large subunit 1	747960.1	27	13	29.31
NP_051066	ATP synthase CF1 beta subunit	411085.9	15	9	25.9
NP_191556	Methylenetetrahydrofolate reductase 1	257774.7	7	5	10.98
NP_198035	Threonyl-tRNA synthetase	403550.4	10	5	10.86
NP_189966	Glutathione S-transferase tau 27	457196.6	17	10	28.63
NP_181684	S-formylglutathione hydrolase	810883	19	9	35.56
NP_196807	Selenoprotein O	754121.8	26	10	8.37
NP_176461	Stress-inducible protein	270221.6	10	6	7.88
NP_199588	Farnesyl diphosphate synthase 1	300497.9	9	4	12.5

NP_195780	Ferretin 1	85073.8	4	3	9.8
NP_001030993	GTP binding Elongation factor Tu family protein	186865.5	8	6	11.58
NP_566473	Subtilase family protein	236228.2	8	5	10.3
NP_176787	NAD(P)-binding Rossmann-fold superfamily protein	216487.9	9	6	17.86
NP_001322980	HISTIDINE TRIAD NUCLEOTIDE-BINDING 2	52338.4	3	2	13.61
NP_181187	Aldolase superfamily protein	138879.3	7	5	18.99
NP_001031969	Glutamine synthetase 2	548201.2	14	9	22.79
NP_001031599	Arginase/deacetylase superfamily protein	187792.6	6	5	19.01
NP_173259	Calcium-binding EF-hand family protein	108325.8	6	5	35.88
NP_566316	Galactose oxidase/kelch repeat superfamily protein	77981.5	5	2	8.21
NP_194664	Profilin 2	167080.5	5	2	9.92
NP_001077529	Glyceraldehyde 3-phosphate dehydrogenase A subunit 2	257311.8	4	3	5.99
NP_566015	Calcium-binding EF-hand family protein	102064.6	4	3	25.35
NP_194713	Pyridoxal-5'-phosphate-dependent enzyme family protein	175863	5	4	10.08
NP_001189932	Presequence protease 1	59284.3	3	3	3.46
NP_190383	Aldehyde dehydrogenase 2B4	420374	10	4	8.18
NP_001324936	Enolase	105715.1	4	2	2.77
NP_565178	Glutathione S-transferase TAU 19	181442.6	7	4	12.79

NP_567952	Cystathionine beta-synthase (CBS) family protein	162299.2	5	3	17.65
NP_193140	Selenium-binding protein 2	97261.7	5	3	6.37
NP_001078356	Stromal ascorbate peroxidase	140099.1	4	3	10.78
NP_199698	Small nuclear ribonucleoprotein family protein	40384.4	2	2	18.18
NP_850070	Nucleotidylyl transferase superfamily protein	128009.8	6	3	6.13
NP_001318975	Pyruvate phosphate dikinase, PEP/pyruvate binding domain-containing protein	256530.4	8	5	5.86
NP_567219	NFU domain protein 1	179212.8	4	3	8.66
NP_568694	Acetoacetyl-CoA thiolase 2	64368.7	2	2	7.69
NP_001184893	Glutathione S-transferase 6	183414.3	9	6	33.17
NP_188056	Pyridoxal-dependent decarboxylase family protein	36365.3	3	2	4.13
NP_200010	GroES-like zinc-binding alcohol dehydrogenase family protein	188133.5	7	4	10.99
NP_194791	Putative BCR, YbaB family COG0718	57291.2	3	2	8.33
NP_172153	Photosystem II subunit P-1	46218.8	4	2	8.75
NP_172508	Glutathione S-transferase family protein	88041.4	5	2	12.33
NP_565666	Copper/zinc superoxide dismutase 2	93119.2	3	2	12.96
NP_190926	Diaminopimelate epimerase family protein	82194.7	3	3	12.15
NP_001321064	Thylakoidal ascorbate peroxidase	38189.8	3	2	5.7
NP_196647	Cystathionine beta-synthase (CBS) family protein	38279.5	3	3	22.33

NP_001078677	pfkB-like carbohydrate kinase family protein	98581.9	4	2	9.06
NP_196867	Magnesium-chelatase subunit chlH, chloroplast, putative / Mg-protoporphyrin IX chelatase, putative (CHLH)	158935	7	5	3.84
NP_178480	Small nuclear ribonucleoprotein family protein	84660.7	3	2	21.21
NP_190400	Aldehyde dehydrogenase 10A9	40854.7	3	3	9.74
NP_200446	Pyruvate kinase family protein	43816.7	2	2	4.02
NP_187678	Non-intrinsic ABC protein 7	98573.1	3	3	14.2
NP_197483	ARM repeat superfamily protein	2518420.7	20	2	0.99
NP_180505	Glutathione S-transferase tau 6	74266.1	2	2	11.21
NP_001318198	Threonyl-tRNA synthetase, putative / threonine-tRNA ligase	333314.6	6	3	6
NP_001077966	Abscisic aldehyde oxidase 3	75155.7	3	3	3.83
NP_195870	Heat shock cognate protein 70-1	75644.6	3	2	1.54
NP_001319970	TIR-NBS-LRR class disease resistance protein	160869.5	3	2	0.97
NP_001320847	Ubiquitin carboxyl-terminal hydrolase-related protein	86703.5	3	2	2.03
NP_174998	Basic region/leucine zipper motif 60	1165346.3	10	2	3.73

Supplementary Table S2. Primers used in this study.

Name	Sequence (5'–3')	Purpose
NdeI-AtCXE5-F	GACCATATGATGGAATCTGAAATCGCC [†]	Cloning
EcoRI-AtCXE5-R	GCTATAGAATTCTCAACCAATAATAAACTCGAC	Cloning
NdeI-AtCXE12-F	GACCATATGGATTCCGAGATCGCCGTC	Cloning
EcoRI-AtCXE12-R	GCTATAGAATTCCTAGTTCCCTCCCTTAATAAAC	Cloning
SAIL-LB	GCCTTTTCAGAAATGGATAAATAGCCTTGCTTCC	Genotyping
SALK-LB	ATTTTGCCGATTTCGGAAC	Genotyping
atcxe12#1-L	CAATGGTACAACCAAACCAAAC	Genotyping
atcxe12#1-R	TCCTGTATCGTTCAAACCAGG	Genotyping
atcxe12#2-L	ATCGAACCAGAATGCATCATC	Genotyping
atcxe12#2-R	TGGAATCATCTTGCTTCATCC	Genotyping
atcxe5-1&2-L	CAATGGTACAACCAAACCAAAC	Genotyping
atcxe5-1&2-R	TCCTGTATCGTTCAAACCAGG	Genotyping
AtCXE5-qF	TGAAGATGTATGGTCCGCGA	RT-PCR
AtCXE5-qR	CAGCTTTCATCGCCATGTGA	RT-PCR
AtCXE12-qF	CAACGTGGTGCAATCAGAGT	RT-PCR
AtCXE12-qR	TCCACCACTTCAACCTCTCC	RT-PCR
AtPP2AA3-qF	GGCAGAAGTTCGGATAGCAG	RT-PCR
AtPP2AA3-qR	CAATGCAGATCTGACGTGCT	RT-PCR

[†] Restriction sites are underlined.

Supplementary Table S3. Plant material used in this study.

Species	Age at use
Arabidopsis thaliana (L.) Heynh.) ecotype Columbia	4-5 weeks
Cucumis sativus L. cv. Marketmore	2 weeks
Helianthus annuus cv. Giganteus	4-5 weeks
Phaseolus vulgaris L. var. *nanus* cv. Highway	2 weeks
Solanum lycopersicum cv. Moneymaker	4-5 weeks
Triticum aestivum cv. Claro	2 weeks
Zea mays subsp. *mays* cv. KN5585	2 weeks
